# [Corrigendum] Lidocaine inhibits the invasion and migration of TRPV6‑expressing cancer cells by TRPV6 downregulation

**DOI:** 10.3892/ol.2023.13957

**Published:** 2023-07-17

**Authors:** Yuan Jiang, Hui Gou, Jiang Zhu, Si Tian, Lehua Yu

Oncol Lett 12: 1164–1170, 2006; DOI: 10.3892/ol.2016.4709

Following the publication of the above article, an interested reader drew to the Editor's attention that, for the micrograph data shown in Fig. 2 on p. 1167, the data panels shown for the ‘PC-3/0 h/0.1 mM Lidocaine’ and ‘PC-3/0 h/1.0 mM Lidocaine’ experiments appeared to be the same; moreover, certain of the data were also found to be overlapping with the ‘PC-3/0 h/0.01 mM Lidocaine’ data panel, such that these data were apparently all derived from the same original source. After having asked the authors to offer an explanation and to re-examine their original data, the authors realized that certain of the data panels for the ‘PC-3/0 h’ experiments had been inadvertently chosen incorrectly.

The revised and corrected version of Fig. 2 is shown on the next page. Note that the errors made during the assembly of this figure did not affect the overall conclusions reported in the paper. All the authors agree with the publication of this corrigendum. The authors are grateful to the Editor of *Oncology Letters* for allowing them the opportunity to publish this, and also apologize to the readership for any inconvenience caused.

## Figures and Tables

**Figure 2.**
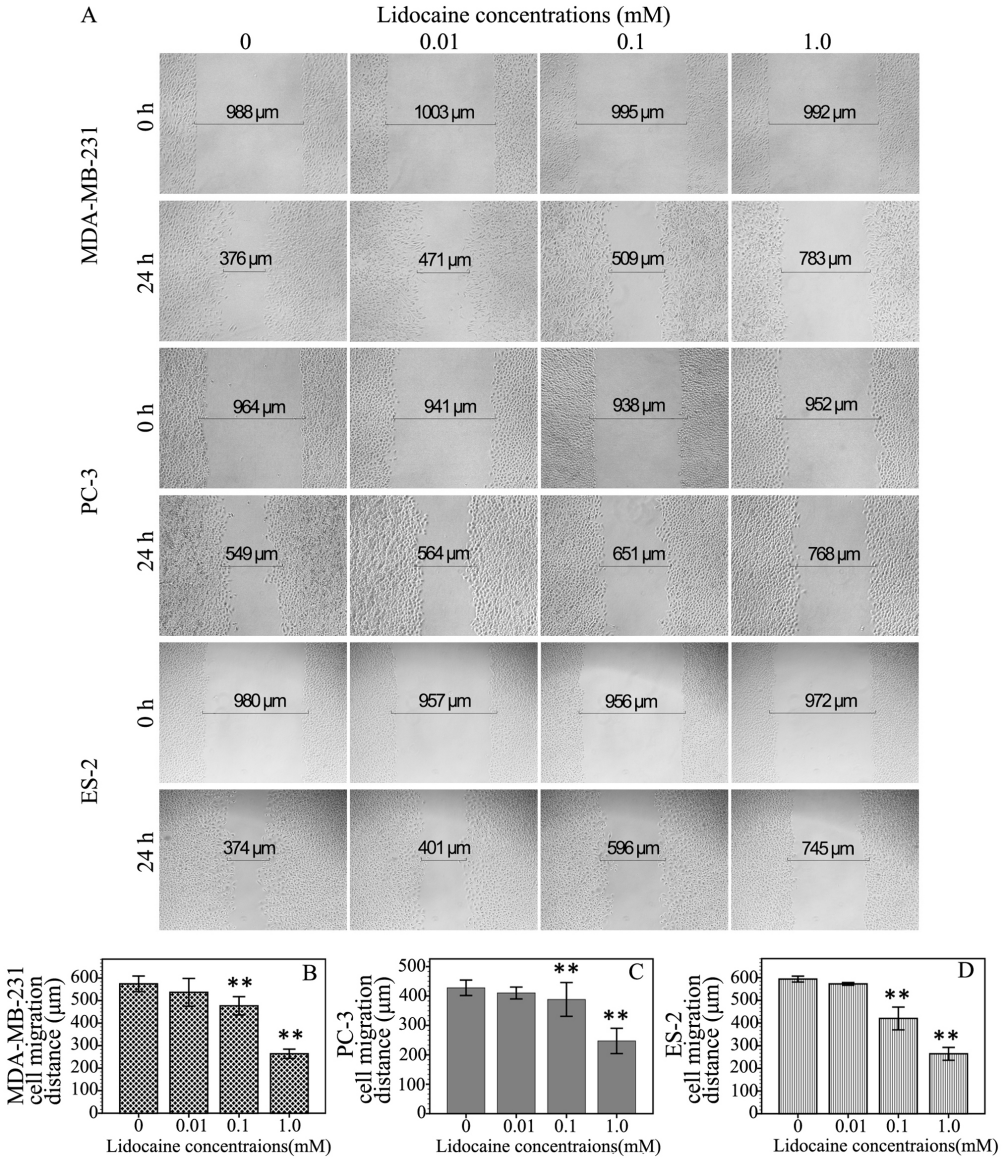
Effect of lidocaine on the cell migration of transient receptor potential cation channel subfamily V member 6-expressing cancer cells. The cells were wounded with a sterile 10-µl plastic micropipette tip to generate a clean scratch wound across the center of the well, and then exposed to different concentrations (0 µM, 10 µM, 100 µM and 1 mM) of lidocaine for 24 h. (A) The migration distance of the cells was evaluated by the wound healing assay at the 0- and 24-h time points (×200 magnification). The histograms represent the migration distance of the (B) MDA-MB-231, (C) PC-3 and (D) ES-2 cells at different concentrations of lidocaine, and the data are shown as the mean ± standard deviation of three independent experiments. *P<0.05 and **P<0.01 vs. control group.

